# Effect of Dotinurad on Uric Acid and Hepatorenal Parameters in Steatotic Liver Disease: A Pilot Study in Japanese Patients

**DOI:** 10.3390/biomedicines13112716

**Published:** 2025-11-05

**Authors:** Yuma Kamijo, Takanobu Iwadare, Takefumi Kimura, Kaede Fujita, Taiki Okumura, Shun-ichi Wakabayashi, Hiroyuki Kobayashi, Tomoo Yamazaki, Naoki Tanaka, Hideo Kunimoto

**Affiliations:** 1 Department of Hepatology, Nagano Municipal Hospital, Nagano 381-8551, Japaniwnb.0522@gmail.com (T.I.); 25hm125a@shinshu-u.ac.jp (K.F.); hideo_kunimoto@hospital.nagano.nagano.jp (H.K.); 2 Department of Medicine, Division of Gastroenterology, Shinshu University School of Medicine, Matsumoto 390-8621, Japan; taiki0960@outlook.jp (T.O.);; 3 Consultation Center for Liver Diseases, Shinshu University Hospital, Matsumoto 390-8621, Japan; 4 Institute for Biomedical Sciences, Research Cluster for Social Implementation, Matsumoto 390-8621, Japan; 5 Department of Global Medical Research Promotion, Shinshu University Graduate School of Medicine, Matsumoto 390-8621, Japan; 6 International Relations Office, Shinshu University School of Medicine, Matsumoto 390-8621, Japan; 7 Research Center for Social Systems, Shinshu University, Matsumoto 390-8621, Japan

**Keywords:** alcohol-related liver disease, dotinurad, hyperuricemia, metabolic dysfunction-associated steatotic liver disease, selective urate transporter 1 modulator, steatotic liver disease

## Abstract

**Background:** Dotinurad (DOT) has demonstrated beneficial metabolic effects in preclinical models as a selective uric acid reabsorption inhibitor. However, its clinical impact on steatotic liver disease (SLD) with hyperuricemia (HU-SLD) remains unclear. **Methods:** This observational pilot study evaluated 33 patients with HU-SLD (Metabolic dysfunction-associated steatotic liver disease: *n* = 20; Metabolic dysfunction-associated alcohol-related liver disease: *n* = 1; Alcohol-related liver disease: *n* = 12) treated with DOT for at least 6 months. Laboratory parameters were assessed at baseline and at 6 months. The primary outcomes were changes in serum uric acid levels, hepatobiliary function markers, and renal function markers. **Results:** DOT significantly reduced serum uric acid levels from 8.4 (7.7–9.0) to 6.0 (5.9–6.8) mg/dL at 6 months (*p* < 0.001). Regarding hepatobiliary markers, gamma-glutamyl transferase decreased from 47 (30–78) to 43 (27–54) U/L (*p* = 0.042) and total bilirubin decreased from 0.6 (0.5–1.0) to 0.6 (0.4–0.7) mg/dL (*p* = 0.023). Significant but modest improvements in renal function were also observed, with serum creatinine decreasing from 1.1 (0.9–1.3) to 1.0 (0.9–1.1) mg/dL (*p* = 0.010) and estimated glomerular filtration rate increasing from 55.6 (44–67.3) to 56.6 (48.8–71.5) mL/min/1.73 m^2^ (*p* = 0.007). No significant changes were observed for aspartate aminotransferase, alanine aminotransferase, fibrosis-related markers, lipid profiles, or glycemic markers. Moreover, no treatment discontinuations or adverse events were recorded during the study period. **Conclusions:** DOT effectively reduced serum uric acid and modestly improved renal and hepatobiliary parameters in HU-SLD without any patient-reported complications. These real-world findings support the potential of DOT as a well-tolerated therapeutic option beyond urate lowering and warrant further investigation in larger, controlled studies.

## 1. Introduction

Steatotic liver disease (SLD) encompasses a spectrum of liver disorders characterized by hepatic fat accumulation, including the conditions traditionally classified as non-alcoholic fatty liver disease (NAFLD) and alcohol-related liver disease (ALD). Recent updates in disease nomenclature have led to the reclassification of NAFLD as metabolic dysfunction-associated steatotic liver disease (MASLD), which requires the presence of at least one cardiometabolic risk factor. As a new category, metabolic dysfunction-associated alcohol-related liver disease (MetALD) has also been proposed to designate patients with both metabolic dysfunction and moderate alcohol consumption [1]. Meanwhile, ALD, which is primarily caused by excessive alcohol consumption, continues to be recognized within the broader category of SLD by reflecting the overlapping pathophysiological mechanisms of metabolic dysfunction, oxidative stress, and inflammatory pathways that contribute to liver injury. Beyond liver-specific outcomes, SLD is strongly associated with an increased risk of extrahepatic complications, including cardiovascular disease, chronic kidney disease, and certain malignancies [2,3,4]. The coexistence of hepatic and renal impairment in SLD has garnered increasing attention due to its clinical implications and potential for targeted intervention [5,6].

Serum uric acid (UA) levels are closely associated with obesity, metabolic syndrome, insulin resistance, and the presence of hepatic steatosis [7,8]. Notably, the prevalence of hyperuricemia (HU) differs across SLD subtypes, being approximately 33–35% in MASLD but only 12–13% in ALD, with MetALD showing intermediate rates [8,9,10]. Approved in Japan in January 2020, dotinurad (DOT) is a novel selective UA reabsorption inhibitor (SURI) with high specificity for urate transporter 1 (URAT1). In preclinical mouse models, DOT has demonstrated beneficial effects on obesity, insulin resistance, and diet-induced hepatic steatosis, as well as potential renoprotective actions [11].

This pilot study evaluated the clinical effects of DOT in patients with SLD accompanied by HU (HU-SLD), including MASLD, MetALD, and ALD forms of the disease.

## 2. Materials and Methods

### 2.1. Patients and Clinical Examinations

**Figure 1:**
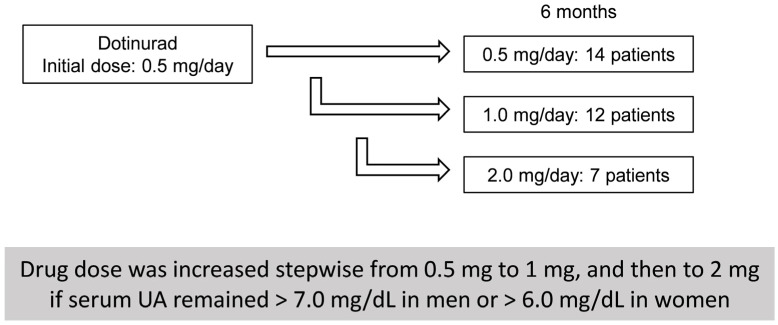
Dotinurad dose escalation and patient distribution at 6 months.

The present retrospective analysis was based on prospectively collected data. This study was approved by Nagano Municipal Hospital (ID number: 0026) and Shinshu University School of Medicine (ID number: 4285), and was performed in adherence to the Helsinki declaration of 1975 (1983 revision). Due to the retrospective study design, the requirement for written informed consent was waived by the respective ethics committees; instead, an opt-out procedure was adopted by disclosing study information on the institutional website to allow patients the opportunity to decline participation. We carefully reviewed the medical records of 33 Japanese SLD patients who had been treated with DOT at Nagano Municipal Hospital (Nagano, Japan) or Shinshu University Hospital (Matsumoto, Japan) between May 2020 and August 2025. The therapeutic effects of DOT administration in HU-SLD patients were investigated using blood test data obtained prior to DOT initiation (baseline) and at 3, 6, and 12 months thereafter. DOT treatment was initiated at 0.5 mg once daily. The dose was increased stepwise from 0.5 mg to 1 mg, and then to 2 mg within 6 months if serum UA remained > 7.0 mg/dL in men or > 6.0 mg/dL in women (Figure 1).

Patients were included if they met the following criteria: (1) hepatic steatosis on abdominal ultrasonography defined as increased hepatic echogenicity relative to the right renal cortex (i.e., hepatorenal contrast) [12]; (2) HU defined as serum UA > 7.0 mg/dL in men and > 6.0 mg/dL in women [13]; and (3) the availability of follow-up data for at least 6 months after the initiation of DOT treatment. The exclusion criteria were alternative causes of liver dysfunction, including viral hepatitis, drug-induced liver injury, autoimmune liver disease, Wilson’s disease, hereditary hemochromatosis, and citrin deficiency [14].

MASLD was defined according to current consensus guidelines as hepatic steatosis in the presence of at least one cardiometabolic risk factor without excessive alcohol consumption [15]. ALD was judged as hepatic steatosis with a history of sustained alcohol consumption ≥ 60 g/day for men and ≥ 50 g/day for women. MetALD was defined as hepatic steatosis in individuals with at least one cardiometabolic risk factor in addition to alcohol consumption above low-risk thresholds (30–60 g/day for men and 20–50 g/day for women) but below the gender-specific amounts considered diagnostic for MASLD [15]. Patients were defined as having hypertension (HT) if their systolic/diastolic pressure was > 140/90 mmHg or if they were taking anti-hypertensive drugs [16]. Type 2 diabetes mellitus (DM) was diagnosed by a fasting plasma glucose level ≥ 126 mg/dL on two occasions, hemoglobin A1c (HbA1c) ≥ 6.5%, plasma glucose ≥ 200 mg/dL at 2 h during an oral glucose tolerance test, or random plasma glucose ≥ 200 mg/dL with classic symptoms of hyperglycemia, or if the patient was taking insulin or oral hypoglycemic agents [17]. Patients were judged as having dyslipidemia (DL) if their fasting serum levels of total cholesterol, low-density lipoprotein cholesterol, or triglyceride (TG) were ≥ 220 mg/dL, ≥ 140 mg/dL, or ≥ 150 mg/dL, respectively, or if they were taking lipid-lowering drugs [18].

All laboratory data were obtained in a fasting state. Albumin-bilirubin (ALBI) score and fibrosis-4 index (FIB-4) were calculated using the following formulae: ALBI score = (log10 total bilirubin [T-Bil] [mg/dL] × 0.66) + (albumin [g/dL] × −0.085 × 10), and FIB-4 = (age [years] × aspartate aminotransferase [AST] [U/L])/(platelet count [PLT] [×10^4^/μL] × 10 × alanine aminotransferase [ALT] [U/L]^1/2^). Blood samples were obtained at baseline as well as at 3, 6, and 12 months of treatment. Regarding adverse events, patients were interviewed at 1–3-month intervals, with relevant information also collected through retrospective chart review.

### 2.2. Statistical Analysis

Clinical data are expressed as the number (percentage) or median (interquartile range [IQR]). Statistical analyses were performed using R software ver. 4.3.0. The Wilcoxon signed-rank test was employed to compare paired continuous variables before and after DOT treatment. Patients were defined as responders if their serum gamma-glutamyl transferase (GGT) levels were decreased ≥30% at 6 months of DOT therapy. The Mann–Whitney U test and Chi-square test were used to compare baseline characteristics between responders and non-responders. To account for multiple comparisons, *p*-values were adjusted using the Bonferroni method. Univariate and multivariate linear regression analyses were performed to identify factors associated with changes in GGT and estimated glomerular filtration rate (eGFR). Candidate variables were first examined individually in univariate analyses, with relevant variables subsequently employed in multivariate models. Stepwise selection based on the Akaike information criterion was applied to determine the final models. Given its clinical relevance, UA was forcibly included as an explanatory variable in multivariate testing. All statistical tests were evaluated at the 0.05 level of significance. Patients with missing laboratory data at key time points were excluded from the corresponding analyses, i.e., no imputation for missing values was performed.

## 3. Results

### 3.1. Clinical Characteristics of HU-SLD Patients Treated with DOT

A total of 33 patients diagnosed with HU-SLD were included in this study (Table 1). Patients were classified into the MASLD, MetALD, or ALD groups based on the respective diagnostic criteria. A total of 60.6% of patients (*n* = 20) were placed into the MASLD group, 3% (*n* = 1) into the MetALD group, and 36.4% (*n* = 12) into the ALD group. Median age was 59 years (IQR: 53–71), and 87.9% of patients were male. During the observation period, no hepatoprotective or nephroprotective agents, such as ursodeoxycholic acid, sodium-glucose cotransporter-2 inhibitors, glucagon-like peptide-1 receptor antagonist, spironolactone, tolvaptan, angiotensin II receptor blockers, angiotensin-converting enzyme inhibitors, or pemafibrate, were co-administered. The prevalence of HT, DM, and DL was 54.5%, 36.4%, and 60.6%, respectively. Among the 33 patients with available anthropometric data, median body weight was 74.3 (65.2–76.6) kg, and median body mass index (BMI) was 24.3 (23.8–29.6) kg/m^2^. In comparisons of baseline characteristics between the ALD and MASLD groups, serum UA levels were significantly higher in the ALD group (9.2 [8.4–9.9] vs. 8.2 [7.4–8.5] mg/dL, *p* = 0.039) (Supplementary Table S1).

### 3.2. Six-Month Outcomes of DOT Treatment in HU-SLD

**Figure 2:**
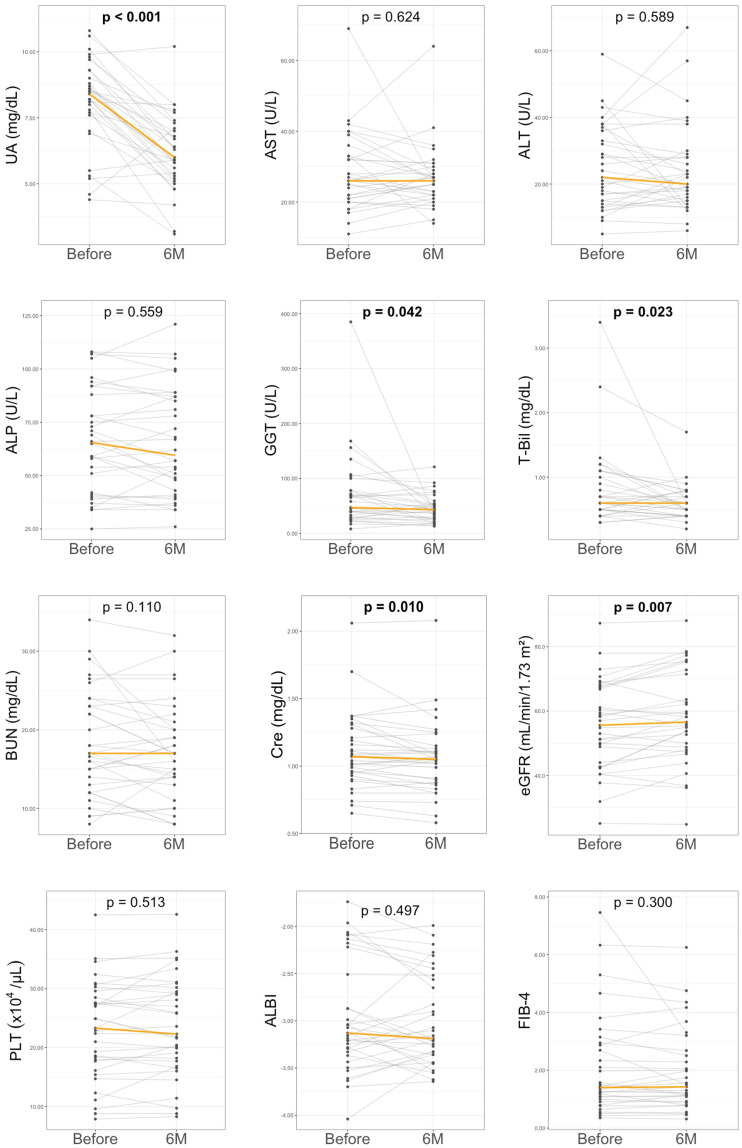
Changes in laboratory parameters before and at 6 months of dotinurad treatment. ALBI, albumin-bilirubin score; ALP, alkaline phosphatase; AST, aspartate aminotransferase; ALT, alanine aminotransferase; BUN, blood urea nitrogen; Cre, creatinine; eGFR, estimated glomerular filtration rate; FIB-4, fibrosis-4 index; GGT, gamma-glutamyltransferase; PLT, platelet count; T-Bil, total bilirubin; UA, uric acid. The orange line represents the median value. Sample size was *n* = 33.

At 6 months of DOT therapy, a significant reduction in UA levels was observed versus baseline (8.4 [7.7–9.0] to 6.0 [5.9–6.8] mg/dL, *p* < 0.001) (Table 1 and Figure 2). No treatment discontinuations due to adverse events were recorded during DOT therapy. In addition to its urate-lowering effect, DOT was associated with an improvement in renal function; serum creatinine levels were significantly decreased (1.1 [0.9–1.3] to 1.0 [0.9–1.1] mg/dL, *p* = 0.010), and eGFR improved from 55.6 (44–67.3) to 56.6 (48.8–71.5) mL/min/1.73 m^2^ (*p* = 0.007). Regarding liver function parameters, DOT treatment led to significant reductions in GGT (47 (30–78) to 43 (27–54) U/L, *p* = 0.042) and T-Bil (0.6 (0.5–1.0) to 0.6 (0.4–0.7) mg/dL, *p* = 0.023) levels. However, no significant changes were noted for liver transaminases (AST or ALT), alkaline phosphatase (ALP), PLT, blood urea nitrogen, lipid parameters, fasting glucose, HbA1c, or fibrosis-related markers including FIB-4, hyaluronic acid, type IV collagen, and autotaxin. No adverse events related to DOT treatment, such as gastrointestinal disorders, arthralgia, or dermatitis, were observed.

### 3.3. Longitudinal Changes in Liver Enzymes and UA by 12-Month DOT Therapy

**Figure 3:**
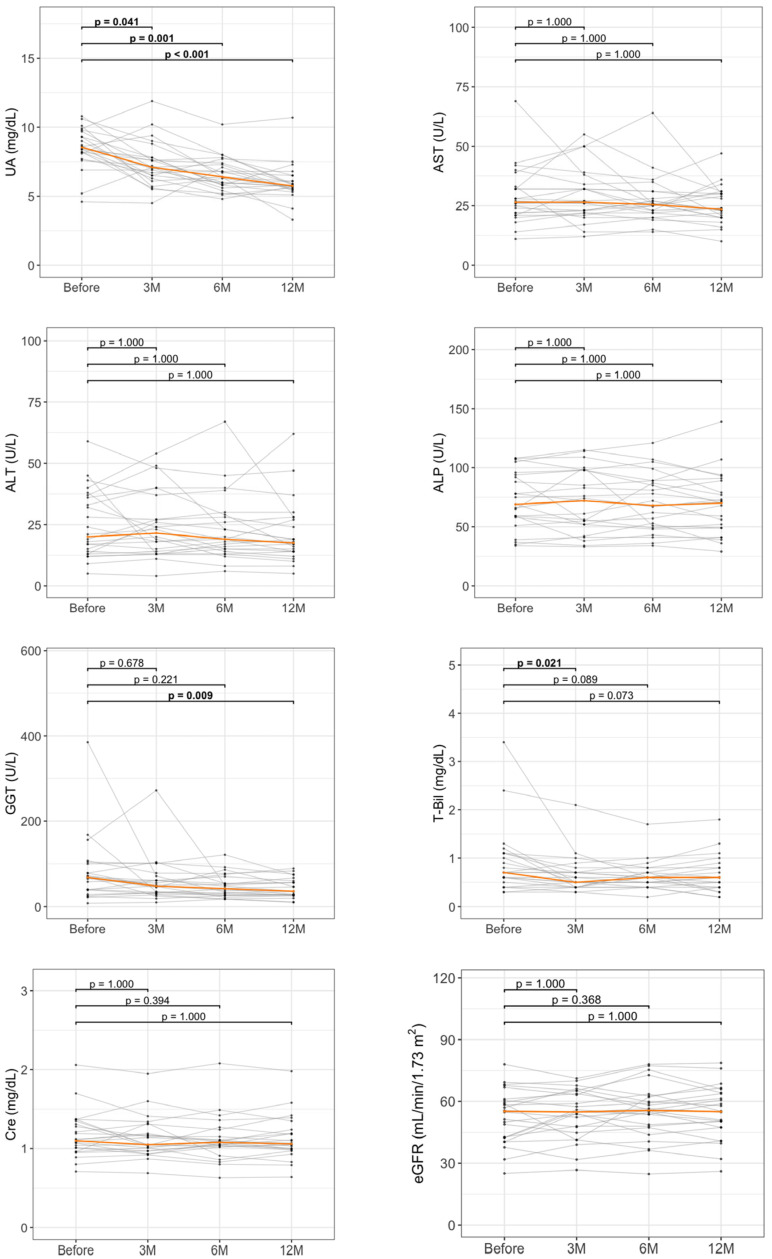
Longitudinal changes in laboratory parameters during dotinurad treatment. Spaghetti plots show changes in UA and liver-related parameters at baseline, 3 months (3M), 6 months (6M), and 12 months (12M). ALP, alkaline phosphatase; ALT, alanine aminotransferase; AST, aspartate aminotransferase; Cre, creatinine; eGFR, estimated glomerular filtration rate; GGT, gamma-glutamyltransferase; M, months; T-Bil, total bilirubin; UA, uric acid. The orange line represents the median value. Sample size was *n* = 24.

To assess the longitudinal effects of DOT, serial measurements of key laboratory parameters were evaluated at baseline and at 3, 6, and 12 months in 24 patients with available blood test data. Multiple comparisons among time points were adjusted using Bonferroni correction. As illustrated in Figure 3, UA levels displayed a significant and sustained decrease beginning at 3 months, with further reductions maintained throughout the 12-month follow-up period (*p* = 0.041 at 3 months, *p* = 0.001 at 6 months, and *p* < 0.001 at 12 months compared with baseline). Liver enzyme levels, including AST, ALT, and ALP, remained relatively unchanged over the treatment course. In contrast, GGT levels progressively decreased, reaching statistical significance at 12 months (*p* = 0.009 vs. baseline). T-Bil levels also exhibited a significant reduction at 3 months (*p* = 0.021 vs. baseline). Renal function parameters showed no significant improvement in this cohort.

### 3.4. Comparison of Clinical Factors According to GGT ≥ 30% Improvement

To investigate the clinical factors associated with changes in GGT levels under DOT treatment, patients were stratified at 6 months into responders (GGT ≥ 30% improvement) and non-responders. At baseline, responders had a significantly higher prevalence of DM (66.7% vs. 19%, *p* = 0.010), as well as higher AST (32.5 (27.2–39.2) vs. 25 (20–27) U/L, *p* = 0.036) and GGT (73.5 (39.8–140.2) vs. 45 (26–67) U/L, *p* = 0.043) levels. No significant differences were observed for other metabolic parameters, including age and BMI (Table 2). Notably, the proportion of responders did not differ significantly between the MASLD (50% vs. 66.7%, *p* = 0.465) and ALD (41.7% vs. 33.3%, *p* = 0.716) groups.

### 3.5. Correlation Between GGT Improvement and Biochemical Parameters

**Figure 4:**
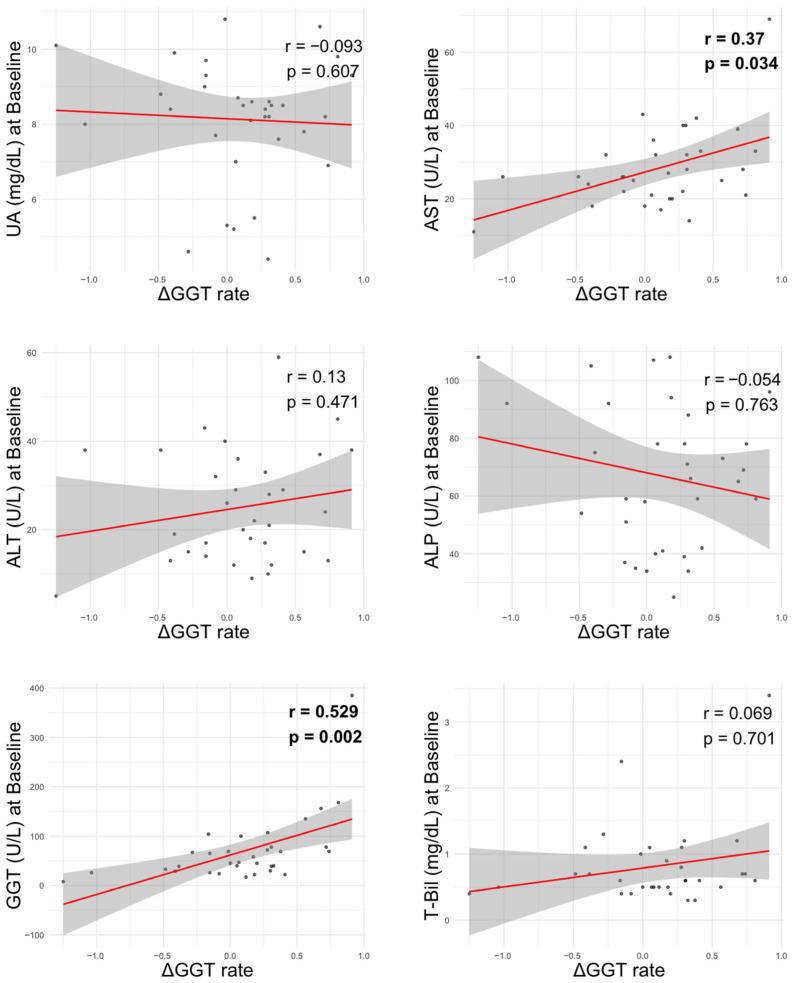
Correlation between liver-related parameters at baseline and GGT improvement rate. ΔGGT rate: ([baseline GGT-6-month GGT]/baseline GGT) ALP, alkaline phosphatase; ALT, alanine aminotransferase; AST, aspartate aminotransferase; GGT, gamma-glutamyltransferase; T-Bil, total bilirubin; UA, uric acid.

To further explore potential factors associated with changes in GGT, we analyzed correlations between the 6-month GGT improvement rate (ΔGGT rate) and clinical laboratory parameters (Figure 4). Among liver-related markers, a significant positive correlation was observed between ΔGGT rate and baseline AST levels (r = 0.37, *p* = 0.034) as well as baseline GGT values (r = 0.529, *p* = 0.002). In contrast, no significant correlations were observed between ΔGGT rate and ALT, ALP, T-Bil, or UA.

### 3.6. Baseline Predictors Changes in GGT and eGFR During DOT Treatment

In univariate analysis, the 6-month changes in GGT were significantly associated with baseline AST (β 0.020, *p* = 0.008) and GGT (β 0.004, *p* < 0.001) levels. In multivariate analysis, ALP (β −0.008, *p* = 0.007), GGT (β 0.005, *p* < 0.001), and BUN (β 0.028, *p* = 0.004) were all independently associated with changes in GGT (Table 3).

Regarding the 6-month changes in eGFR, univariate analysis revealed a significant association with baseline albumin (β −0.004, *p* = 0.003). In multivariate analysis, AST (β −0.011, *p* = 0.002) and PLT (β −0.006, *p* = 0.018) were independently associated with changes in eGFR (Table 4).

## 4. Discussion

### 4.1. Main Findings

This study evaluated the impact of DOT on liver and kidney function in patients with HU-SLD. DOT treatment led to significant improvements in serum UA and GGT levels. Furthermore, DOT therapy was associated with a modest, but significant, amelioration in renal function as evidenced by changes in creatinine and eGFR levels. These favorable changes in hepatobiliary and renal parameters were observed regardless of alcohol consumption, suggesting a potential therapeutic effect independent of disease etiology. No treatment discontinuations or adverse drug-related events were recorded during the observation period, which demonstrated the safety of DOT.

### 4.2. Context with Published Literature

In the kidney, DOT acts on URAT1 in the proximal tubules by lowering intracellular UA and promoting urinary UA excretion without significant inhibition of other urate transporters, including glucose transporter 9 (GLUT9), ATP-binding cassette transporter G2 (ABCG2), and organic anion transporter 1/3 (OAT1/3) [19]. By avoiding such inhibition, DOT may minimize off-target metabolic effects and drug–drug interactions. Reduced intracellular UA may limit oxidative stress, endothelial dysfunction, and activation of the NOD-like receptor family pyrin domain-containing 3 (NLRP3) inflammasome [20]. Both in its soluble form and as monosodium urate crystals, UA can act in a damage-associated molecular pattern by triggering NLRP3 activation, IL-1β release, and downstream inflammatory responses, thereby contributing to tissue injury [20]. Recent clinical studies have also demonstrated that DOT significantly reduces serum UA levels and helps preserve renal function across multiple cohorts (Table 5) [21,22,23,24,25]. In line with these reports, our study confirmed a robust reduction in serum UA levels along with modest, but significant, improvements in renal markers during DOT therapy in patients with HU-SLD.

**Figure 5:**
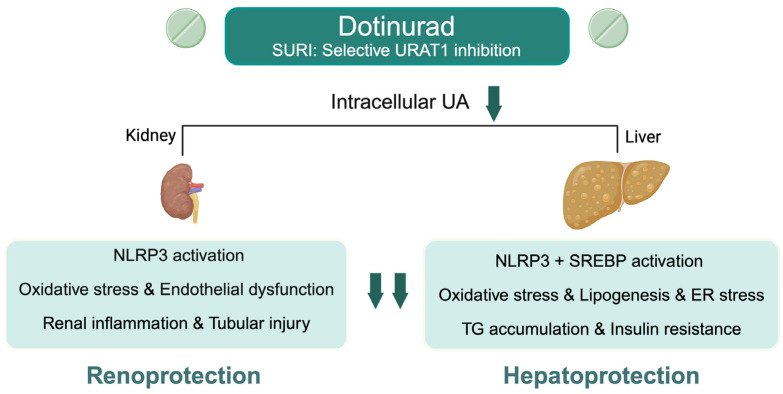
Proposed molecular mechanisms of renal and hepatic protection by dotinurad through selective URAT1 inhibition in hyperuricemia-associated steatotic liver disease. ER, endoplasmic reticulum; NLRP3, NOD-like receptor family pyrin domain-containing 3; SREBP, sterol regulatory element-binding protein; TG, triglyceride; UA, uric acid; URAT1, urate transporter 1. Created in BioRender. Kimura, T. (2025) https://BioRender.com/gmchkc8.

In the liver and adipose tissue, preclinical studies have shown that URAT1 inhibition ameliorates steatosis, insulin resistance, and mitochondrial dysfunction largely through reductions in oxidative stress, lipogenesis, and pro-inflammatory signaling [11]. UA is also a known activator of the NLRP3 inflammasome, promoting hepatic inflammation and fibrosis via IL-1β and IL-18 release as well as pyroptotic cell death [26]. Additional UA-related mechanisms include endoplasmic reticulum stress-induced activation of sterol regulatory element-binding proteins (SREBP), which leads to lipid accumulation and insulin resistance [27,28]. Our findings of improved GGT without significant improvements in AST or ALT support these experimental observations, suggesting a beneficial contribution to oxidative stress and cholestasis [29]. Biologically, UA lowering may preferentially mitigate oxidative stress and cholestatic pathways rather than hepatocellular injury, which helps explain the greater effect on GGT compared with AST and ALT. Based on the above lines of reasoning (Figure 5), our clinical results demonstrating improvements in both renal and hepatobiliary parameters with DOT lend indirect support to several mechanistic pathways and highlight the potential of urate-lowering therapies in SLD.

### 4.3. Strengths and Limitations

A key strength of this study is the longitudinal assessment of liver- and renal-related biomarkers over a 12-month period, offering insights into treatment dynamics over time. The inclusion of patients with MASLD, MetALD, and ALD enhances the generalizability of the findings as well. To our knowledge, this is among the first clinical reports to suggest potential hepatoprotective effects of DOT in SLD patients, particularly on GGT, alongside its known UA lowering action.

However, several limitations should be noted. This was a retrospective observational study with a small sample size and no control group, which limited causal inferences. Some analyses were based on reduced subsamples, which weakened robustness and generalizability. Nutritional and lifestyle factors, including diet and physical activity, were not systematically assessed [30,31]. Furthermore, attrition bias was possible due to incomplete follow-up and missing data. Finally, no histological or imaging assessments (e.g., liver biopsy, FibroScan, or MRI-PDFF) were available to validate the changes in steatosis or fibrosis.

### 4.4. Future Implications

The current findings suggest that DOT, beyond its primary indication for HU, may also confer hepatorenal-protective effects in patients with SLD. To validate these results, additional prospective, randomized controlled trials are warranted in other ethnicities. Such studies should evaluate hepatic and metabolic outcomes using endpoints such as MRI-PDFF, transient elastography, and histological assessment. Given its favorable safety profile and potential hepatorenal benefits, DOT may represent a promising treatment option for patients with HU-SLD.

## Figures and Tables

**Table 1:** Comparison of Clinical and Laboratory Data Before and at 6 Months of Dotinurad Therapy (*n* = 33).

	Before	6 Months	*p*-Value
Age (years)	59 (53–71)	-	-
Male (%)	29 (87.9%)	-	-
HT (%)	18 (54.5%)	-	-
DM (%)	12 (36.4%)	-	-
DL (%)	20 (60.6%)	-	-
MASLD (%)	20 (60.6%)	-	-
MetALD (%)	1 (3%)	-	-
ALD (%)	12 (36.4%)	-	-
Body weight (kg)	74.3 (65.2–76.6)	72.2 (65.4–77.6)	0.221
BMI (kg/m^2^)	24.3 (23.8–29.6)	26.9 (24.6–30.3)	0.678
ALB (g/dL)	4.5 (3.8–4.8)	4.4 (3.8–4.7)	0.844
AST (U/L)	26 (21–33)	26 (22–29)	0.623
ALT (U/L)	22 (15–36)	20 (15–28)	0.589
ALP (U/L)	65.5 (41.8–89)	59.5 (42.5–87)	0.559
**GGT (U/L)**	**47 (30–78)**	**43 (27–54)**	**0.042**
**T-Bil (mg/dL)**	**0.6 (0.5–1.0)**	**0.6 (0.4–0.7)**	**0.023**
PLT (×10^4^/μL)	23.3 (17.7–28.5)	22.3 (17.7–29.5)	0.513
BUN (mg/dL)	17 (13–23)	17 (14–20)	0.110
**Cre (mg/dL)**	**1.1 (0.9–1.3)**	**1.0 (0.9–1.1)**	**0.010**
**eGFR (mL/min/1.73 m^2^)**	**55.6 (44–67.3)**	**56.6 (48.8–71.5)**	**0.007**
TC (mg/dL)	191 (164–218)	190 (158–209)	0.069
TG (mg/dL)	144 (89–169)	123 (80–170)	0.706
LDL-C (mg/dL)	119 (89–127.2)	110 (83.8–126.8)	0.198
HDL-C (mg/dL)	49.5 (41.5–54.8)	51 (45.2–55.8)	0.230
BS (mg/dL)	123 (107.5–178)	126.5 (118–134)	0.964
HbA1c (%)	6.2 (5.9–7.0)	6.1 (5.9–6.8)	0.611
**UA (mg/dL)**	**8.4 (7.7–9.0)**	**6.0 (5.9–6.8)**	**<0.001**
ALBI score	−3.1 (−3.3 to −2.5)	−3.2 (−3.4 to −2.6)	0.497
FIB-4 index	1.4 (1.0–2.9)	1.4 (0.9–2.5)	0.300
ATX (mg/L)	1.1 (1.0–1.2)	1.0 (0.7–1.1)	0.683
HA (ng/mL)	23.5 (10.2–81.1)	36.8 (10.2–75.1)	0.683
Type IV collagen (ng/mL)	3.8 (3.5–3.9)	3.5 (3.5–3.8)	0.529

ALB, albumin; ALBI, albumin-bilirubin; ALD, alcohol-related liver disease; ALP, alkaline phosphatase; ALT, alanine aminotransferase; AST, aspartate aminotransferase; ATX, autotaxin; BMI, body mass index; BS, blood sugar; BUN, blood urea nitrogen; Cre, creatinine; DM, diabetes mellitus; DL, dyslipidemia; eGFR, estimated glomerular filtration rate; FIB-4, fibrosis-4; GGT, gamma-glutamyltransferase; HA, hyaluronic acid; HbA1c, hemoglobin A1c; HDL-C, high-density lipoprotein cholesterol; HT, hypertension; LDL-C, low-density lipoprotein cholesterol; MASLD; metabolic dysfunction-associated steatotic liver disease; MetALD, metabolic dysfunction-associated alcohol-related liver disease; PLT, platelet count; T-Bil, total bilirubin; TC, total cholesterol; TG, triglyceride; UA, uric acid. Sample size was *n* = 33.

**Table 2:** Comparison of Clinical and Laboratory Data Before and at 6 Months of Dotinurad Therapy.

	Responders (*n* = 12)	Non-Responders (*n* = 21)	*p*-Value
Age (years)	60 (52.2–71.8)	59 (53–71)	0.881
Male (%)	9 (75%)	20 (95.2%)	0.364
HT (%)	7 (58.3%)	11 (52.4%)	1.000
**DM (%)**	**8 (66.7%)**	**4 (19%)**	**0.010**
DL (%)	8 (66.7%)	12 (57.1%)	0.719
MASLD (%)	6 (50%)	14 (66.7%)	0.465
ALD (%)	5 (41.7%)	7 (33.3%)	0.716
BMI (kg/m^2^)	24.4 (22.8–26.9)	26.6 (23.1–29.6)	0.673
ALB (g/dL)	4.4 (4.2–4.6)	4.5 (3.6–4.8)	0.822
**AST (U/L)**	**32.5 (27.2–39.2)**	**25 (20–27)**	**0.036**
ALT (U/L)	26 (14.5–37.2)	20 (15–33)	0.613
ALP (U/L)	67.5 (59–74.2)	59 (40–92)	0.940
**GGT (U/L)**	**73.5 (39.8–140.2)**	**45 (26–67)**	**0.043**
T-Bil (mg/dL)	0.6 (0.6–0.8)	0.6 (0.5–1.0)	0.836
PLT (×10^4^/μL)	22.9 (18.3–27.8)	24.9 (15.4–29.6)	1.000
BUN (mg/dL)	18 (14.2–24.6)	16 (13–23)	0.431
Cre (mg/dL)	1.0 (0.9–1.1)	1.1 (1.0–1.3)	0.184
eGFR (mL/min/1.73 m^2^)	59.1 (47.9–67.8)	54.8 (44–66.8)	0.837
TC (mg/dL)	197 (153–223)	186 (161.5–204.8)	0.869
TG (mg/dL)	140 (88.5–188)	152 (91–169)	1.000
LDL-C (mg/dL)	115 (85–131.5)	122.5 (104.2–133.8)	0.288
HDL-C (mg/dL)	54.5 (42–58.8)	45.5 (41–49.8)	0.142
BS (mg/dL)	140.5 (124–162)	111 (103–139.5)	0.232
HbA1c (%)	6.0 (5.9–6.5)	5.8 (5.4–6.4)	0.326
UA (mg/dL)	8.3 (7.8–8.8)	8.4 (7.7–9)	0.896

Responders: patients with a reduction in GGT levels ≥ 30% at 6 months of dotinurad therapy. Non-responders: patients without a reduction in GGT levels of ≥ 30% at 6 months of dotinurad therapy. ALB, albumin; ALD, alcohol-related liver disease; ALP, alkaline phosphatase; ALT, alanine aminotransferase; AST, aspartate aminotransferase; BMI, body mass index; BS, blood sugar; BUN, blood urea nitrogen; Cre, creatinine; DM, diabetes mellitus; DL, dyslipidemia; eGFR, estimated glomerular filtration rate; GGT, gamma-glutamyltransferase; HbA1c, hemoglobin A1c; HDL-C, high-density lipoprotein cholesterol; HT, hypertension; LDL-C, low-density lipoprotein cholesterol; MASLD, metabolic dysfunction associated steatotic liver disease; PLT, platelet count; T-Bil, total bilirubin; TC, total cholesterol; TG, triglyceride; UA, uric acid. Sample size was *n* = 33.

**Table 3:** Baseline Factors Associated with Changes in GGT: Univariate and Multivariate Analyses.

	Univariate Analysis			Multivariate Analysis		
	β	95% CI	*p*-Value	β	95% CI	*p*-Value
Age	0.005	(−0.007–0.017)	0.372			
Male	−0.208	(−0.731–0.314)	0.422	−0.344	(−0.744–0.056)	0.088
HT	0.135	(−0.208–0.477)	0.429			
DM	0.327	(−0.011–0.664)	0.058			
DL	0.007	(−0.346–0.359)	0.969			
MASLD	−0.070	(−0.421–0.282)	0.689			
ALD	0.012	(−0.346–0.370)	0.947			
BMI	−0.009	(−0.046–0.029)	0.644			
ALB	0.160	(−0.13–0.45)	0.268			
AST	**0.020**	**(0.006–0.034)**	**0.008**			
ALT	0.007	(−0.007–0.021)	0.302	−0.011	(−0.024 to −0.001)	0.078
ALP	−0.004	(−0.011–0.003)	0.282	**−0.008**	**(−0.013 to −0.002)**	**0.007**
GGT	**0.004**	**(0.002–0.006)**	**<0.001**	**0.005**	**(0.003–0.007)**	**<0.001**
T-Bil	0.170	(−0.106–0.447)	0.218			
PLT	−0.008	(−0.028–0.013)	0.459			
BUN	0.018	(−0.007–0.044)	0.143	**0.028**	**(0.010–0.047)**	**0.004**
Cre	−0.001	(−0.619–0.616)	0.996			
eGFR	−0.002	(−0.015–0.01)	0.697			
UA	−0.016	(−0.123–0.092)	0.77	−0.047	(−0.130–0.035)	0.247

ALB, albumin; ALD, alcohol-related liver disease; ALP, alkaline phosphatase; ALT, alanine aminotransferase; AST, aspartate aminotransferase; BMI, body mass index; BUN, blood urea nitrogen; Cre, creatinine; DM, diabetes mellitus; DL, dyslipidemia; eGFR, estimated glomerular filtration rate; GGT, gamma-glutamyltransferase; HT, hypertension; MASLD, metabolic dysfunction associated steatotic liver disease; PLT, platelet count; T-Bil, total bilirubin; UA, uric acid.

**Table 4:** Baseline Factors Associated with Changes in eGFR: Univariate and Multivariate Analyses.

	Univariate Analysis			Multivariate Analysis		
	β	95% CI	*p*-Value	β	95% CI	*p*-Value
Age	0.000	(−0.003–0.002)	0.853			
Male	0.047	(−0.059–0.152)	0.377	−0.123	(−0.249–0.002)	0.054
HT	0.024	(−0.046–0.094)	0.488			
DM	−0.010	(−0.083–0.062)	0.771	−0.054	(−0.120–0.011)	0.100
DL	0.009	(−0.062–0.081)	0.789	0.072	(−0.002–0.147)	0.055
MASLD	0.017	(−0.054–0.088)	0.628			
ALD	−0.020	(−0.092–0.053)	0.584			
BMI	−0.051	(−0.108–0.006)	0.079			
ALB	**−0.004**	**(−0.007 to −0.002)**	**0.003**	−0.057	(−0.119–0.005)	0.069
AST	−0.002	(−0.005–0.001)	0.129	**−0.011**	**(−0.017 to −0.005)**	**0.002**
ALT	0.000	(−0.001–0.002)	0.567	0.002	(−0.001–0.006)	0.156
ALP	0.000	(−0.001–0.000)	0.173			
GGT	−0.029	(−0.086–0.027)	0.299	0.001	(0.000–0.002)	0.066
T-Bil	−0.002	(−0.007–0.002)	0.253			
PLT	−0.003	(−0.008–0.002)	0.253	**−0.006**	**(−0.011 to −0.001)**	**0.018**
BUN	−0.048	(−0.173–0.076)	0.432			
Cre	0.001	(−0.001–0.004)	0.273			
eGFR	0.002	(−0.02–0.024)	0.826			
UA	−0.051	(−0.108–0.006)	0.079	0.008	(−0.012–0.029)	0.393

ALB, albumin; ALD, alcohol-related liver disease; ALP, alkaline phosphatase; ALT, alanine aminotransferase; AST, aspartate aminotransferase; BMI, body mass index; BUN, blood urea nitrogen; Cre, creatinine; DM, diabetes mellitus; DL, dyslipidemia; eGFR, estimated glomerular filtration rate; GGT, gamma-glutamyltransferase; HT, hypertension; MASLD, metabolic dysfunction associated steatotic liver disease; PLT, platelet count; T-Bil, total bilirubin; UA, uric acid.

**Table 5:** Summary of Recent Clinical Data on Dotinurad.

Author	No	Study Design	Pre UA (mg/dL)	3M UA (mg/dL)	6M UA (mg/dL)	Pre eGFR (mL/min/1.73 m^2^)	3M eGFR (mL/min/1.73 m^2^)	6M eGFR (mL/min/1.73 m^2^)
Tanaka, et al. 2023 [21]	50	Prospective	8.3	5.2	5.2	47.8	47.2	46.9
Amano, et al. 2024 [22]	35	Retrospective	8.1	6.7	-	31.8	35.5	-
Motomura, et al. 2025 [23]	14	Retrospective	8.2	-	6.2	24.9	-	24.4
Takata, et al. 2025 [24]	29	Retrospective	8.4	6.5	-	33.9	36.2	-
Yanai, et al. 2025 [25]	73	Retrospective	6.8	-	5.8	61.2	-	59.2
Present study	33	Retrospective	8.4	7.1	6.0	55.6	56.8	56.6

eGFR, estimated glomerular filtration rate; M, month; No, number; UA, uric acid.

## Data Availability

The data supporting the findings of this study are not publicly available due to privacy and ethical restrictions but are available from the corresponding author upon reasonable request.

## Supplementary Materials

The following supporting information can be downloaded at: https://www.mdpi.com/article/10.3390/biomedicines13112716/s1, Supplementary Table S1 Clinical and Laboratory Data at Baseline in ALD and MASLD Groups.

## Abbreviations

ALBI, albumin-bilirubin; ALD, alcohol-related liver disease; ALP, alkaline phosphatase; ALT, alanine aminotransferase; AST, aspartate aminotransferase; BMI, body mass index; DM, diabetes mellitus; DL, dyslipidemia; DOT, dotinurad; eGFR, estimated glomerular filtration rate; FIB-4, fibrosis-4 index; GGT, gamma-glutamyl transferase; HbA1c, hemoglobin A1c; HT, hypertension; HU, hyperuricemia; HU-SLD, hyperuricemia-associated steatotic liver disease; IQR, interquartile range; MASLD, metabolic dysfunction-associated steatotic liver disease; MetALD, metabolic dysfunction-associated alcohol-related liver disease; MRI-PDFF, magnetic resonance imaging proton density fat fraction; NAFLD, non-alcoholic fatty liver disease; NLRP3, NOD-like receptor family pyrin domain-containing 3; PLT, platelet count; SLD, steatotic liver disease; SURI, selective uric acid reabsorption inhibitor; T-Bil, total bilirubin; TG, triglyceride; UA, uric acid; URAT1, urate transporter 1; ΔGGT rate, GGT improvement rate.
