# Supplementary material for: Effect of Dotinurad on Uric Acid and Hepatorenal Parameters in Steatotic Liver Disease: A Pilot Study in Japanese Patients
Source: Biomedicines. 2025 Nov 5;13(11):2716. doi: 10.3390/biomedicines13112716 (PMC12650707; doi:10.3390/biomedicines13112716)
Supplement: Supplementary file 1 [file biomedicines-13-02716-s001.zip › biomedicines-3797941-supplementary.pdf]

Supplementary

Table S1. Clinical and Laboratory Data at Baseline in ALD and MASLD Groups.

	ALD (n = 12)	MASLD (n = 20)	p-value
Age (years)	59 (53–70.2)	61 (50.8–71.2)	1.000
Male (%)	11 (92%)	17 (85%)	1.000
HT (%)	6 (50%)	12 (60%)	0.718
DM (%)	4 (33%)	8 (40%)	1.000
DL (%)	6 (50%)	14 (70%)	0.288
Body weight (kg)	73.7 (62.4–76.4)	75 (60–84.7)	0.344
BMI (kg/m^2)	24.3 (23.6–26.8)	26.8 (22.9–31)	0.292
ALB (g/dL)	4.4 (3.6–4.7)	4.5 (4.2–4.8)	0.755
AST (U/L)	30.5 (23.5–40)	26 (20–32)	0.119
ALT (U/L)	27 (12.8–38.5)	23 (16.5–29.8)	0.785
ALP (U/L)	74.5 (58.8–98.2)	59 (39.8–78)	0.102
GGT (U/L)	73.5 (37.5–119.2)	42.5 (26–67.5)	0.054
T-Bil (mg/dL)	1.0 (0.6–1.1)	0.6 (0.5–0.7)	0.068
PLT ($\times 10^4/\mu L$)	22.9 (12–27.1)	23.9 (17.9–28.8)	0.483
BUN (mg/dL)	18.5 (13.8–22.2)	16.8 (13.8–24.5)	0.815
Cre (mg/dL)	1.1 (1–1.1)	1.1 (0.9–1.3)	0.953
eGFR (mL/min/1.73 m^2)	53 (49.5–59.7)	56.3 (42.1–68.5)	0.654
TC (mg/dL)	199 (179–231)	185 (143–198.5)	0.127
TG (mg/dL)	153 (97–173.5)	142 (85.5–175.5)	0.606
LDL-C (mg/dL)	135.5 (115–157.5)	116.5 (87–126)	0.107
HDL-C (mg/dL)	48 (45–63)	47.5 (41–53.5)	0.576
BS (mg/dL)	109 (103–138)	126 (107–158)	0.695
**UA (mg/dL)**	**9.2 (8.4–9.9)**	**8.2 (7.4–8.5)**	**0.039**
ALBI score	-3.0 (-3.3 to -2.2)	-3.2 (-3.3 to -3.0)	0.988
FIB-4 index	1.8 (1.2–3.3)	1.4 (0.8–2.5)	0.235

ALB; albumin, ALBI; albumin-bilirubin, ALD; alcohol-related liver disease, ALP; alkaline phosphatase, ALT; alanine aminotransferase, AST; aspartate aminotransferase, BMI; body mass index, BS; blood sugar, BUN; blood urea nitrogen, Cre; creatinine, DM; diabetes mellitus, DL; dyslipidemia, eGFR; estimated glomerular filtration rate, FIB-4; fibrosis-4, GGT; gamma-glutamyltransferase, HDL-C; high-density lipoprotein cholesterol, HT; hypertension, LDL-C; low-density lipoprotein cholesterol, MASLD; metabolic dysfunction associated steatotic liver disease, PLT; platelet count, T-Bil; total bilirubin, TC; total cholesterol, TG; triglyceride, UA; uric acid.
